# Transcranial Magnetic Stimulation of the Dorsolateral Prefrontal Cortex Increases Posterior Theta Rhythm and Reduces Latency of Motor Imagery

**DOI:** 10.3390/s23104661

**Published:** 2023-05-11

**Authors:** Semen Kurkin, Susanna Gordleeva, Andrey Savosenkov, Nikita Grigorev, Nikita Smirnov, Vadim V. Grubov, Anna Udoratina, Vladimir Maksimenko, Victor Kazantsev, Alexander E. Hramov

**Affiliations:** 1 Baltic Center for Neurotechnology and Artificial Intelligence, Immanuel Kant Baltic Federal University, 236016 Kaliningrad, Russia; andrey.savosenkov@gmail.com (A.S.); na0grigorev@gmail.com (N.G.); nikita.smirnov.m@gmail.com (N.S.); vvgrubov@gmail.com (V.V.G.); maximenkovl@gmail.com (V.M.); hramovae@gmail.com (A.E.H.); 2 Neurodynamics and Cognitive Technology Laboratory, Lobachevsky State University of Nizhny Novgorod, 603105 Nizhniy Novgorod, Russia; gordleeva@neuro.nnov.ru (S.G.); a.udoratina@gmail.com (A.U.); kazantsev@neuro.nnov.ru (V.K.)

**Keywords:** motor imagery, transcranial magnetic stimulation, sensorimotor integration, motor imagery latency, dorsolateral prefrontal cortex, precuneus

## Abstract

Experiments show activation of the left dorsolateral prefrontal cortex (DLPFC) in motor imagery (MI) tasks, but its functional role requires further investigation. Here, we address this issue by applying repetitive transcranial magnetic stimulation (rTMS) to the left DLPFC and evaluating its effect on brain activity and the latency of MI response. This is a randomized, sham-controlled EEG study. Participants were randomly assigned to receive sham (15 subjects) or real high-frequency rTMS (15 subjects). We performed EEG sensor-level, source-level, and connectivity analyses to evaluate the rTMS effects. We revealed that excitatory stimulation of the left DLPFC increases theta-band power in the right precuneus (PrecuneusR) via the functional connectivity between them. The precuneus theta-band power negatively correlates with the latency of the MI response, so the rTMS speeds up the responses in 50% of participants. We suppose that posterior theta-band power reflects attention modulation of sensory processing; therefore, high power may indicate attentive processing and cause faster responses.

## 1. Introduction

Motor imagery (MI) is defined as the mental simulation of a given movement in working memory without any overt motor output [1]. MI is widely used motor and as a technique in neurorehabilitation [2,3,4,5,6,7], motor and skill training [8,9,10,11]. Although the contribution of MI to the improvement of motor performance is well established [12], the neural mechanisms underlying MI are not fully understood.

Neuroimaging studies reveal that MI consistently recruits a network of bilateral premotor (supplementary motor areas, the dorsal and ventral premotor cortices, the cingulate and putamen), rostral inferior and middle superior parietal (the inferior and superior parietal lobules), basal ganglia, and cerebellar regions [13,14,15,16].

A recent meta-analysis identified the involvement of the left dorsolateral prefrontal cortex (DLPFC) in MI rather than in movement execution or motor observation [15]. Nevertheless, the functional contribution of the DLPFC to MI is still undefined, impair th giving rise to several hypotheses [17]. It is assumed that involvement of this area in MI may be due to the necessity of working memory activation during MI [18,19] or the similarity of MI to frontal-executive functions related to action preparation [20]. Alternatively, as the role of DLPFC in movement inhibition is well established [21,22,23], it is supposed that DLPFC activation in MI tasks reflects control of overt movement prevention.

In line with these hypotheses, numerous studies have shown a greater involvement of DLPFC in MI than in motor execution [24,25,26,27,28,29,30]. Kim et al. showed that connectivity between the premotor cortex and DLPFC is crucial for performing MI [31]. Moreover, the connectivity strength from the supplementary motor area to the DLPFC is positively correlated with MI performance [32,33]. Mizuguchi et al. found that the right DLPFC and the right inferior parietal lobule showed a higher level of activation during MI while holding an object [34].

Transcranial magnetic stimulation (TMS) is a non-invasive technique that uses magnetic fields to stimulate specific regions of the brain. TMS allows for the direct modulation of neural activity in targeted brain regions, providing insights into their causal role in cognitive processes [35,36]. It has been widely used in neuroscience research to investigate the involvement of different brain areas in various cognitive processes, including MI. TMS studies focusing on MI have aimed to identify the brain regions involved in this process and understand the underlying neural mechanisms. Specifically, TMS applied over the primary motor cortex (M1) during MI tasks has been shown to modulate corticospinal excitability, as measured by motor-evoked potentials recorded from muscles [37,38,39]. This suggests that M1 plays a crucial role in MI processes. Also, TMS studies have shown that stimulating the supplementary motor area (SMA) during MI tasks can affect response times, accuracy, and motor cortex excitability [40,41]. These findings suggest that the SMA contributes to MI by coordinating and planning motor sequences. Furthermore, TMS studies have revealed that stimulating the dorsal premotor cortex (PMd) during MI tasks can modulate corticospinal excitability [42,43]. This shows that the PMd is involved in motor planning and execution processes during MI. Also, the parietal cortex has been implicated in sensorimotor integration and spatial processing during motor imagery. TMS studies have shown that disrupting the parietal cortex during MI tasks can impair performance [44,45,46], suggesting its involvement in motor imagery-related processes. Interestingly, Martel and Glover have shown recently that inhibitory TMS over the right DLPFC slowed MI without affecting overt actions, suggesting a specific role for this region in the control of movement inhibition during MI tasks [17].

The mentioned studies, among many others, highlight the utility of TMS in elucidating the causal role of specific brain areas during MI. However, it is worth noting that the interpretation of TMS results requires careful consideration, and additional research is often necessary to fully understand the complex interplay of brain networks involved in cognitive processes. Particularly, the functional role of the DLPFC in MI tasks requires further investigation.

The present study aimed to investigate the functional contribution of the left DLPFC to the MI using transcranial magnetic stimulation (TMS). We applied a single session of high-frequency repetitive TMS (rTMS) to the left DLPFC before healthy subjects started MI tasks. We hypothesized that rTMS would alter neurophysiological correlates of MI (e.g., the characteristics of EEG event-related desynchronization (ERD) [47,48]). We analyzed the effects of rTMS on the neural source power distributions and functional connectivity between the left DLPFC and other areas involved in the MI task. Moreover, we estimated how rTMS influences the performance of MI using the latency of the MI-related ERD. This approach will allow us to gain insights into the neurophysiological correlates of MI and the specific contribution of the left DLPFC to this cognitive process.

## 2. Materials and Methods

### 2.1. Subjects

Thirty right-handed healthy adult volunteers (21 females/9 males; age range: 18–34 years old; age (mean ± standard deviation (SD)): 20.93 ± 2.14 years) with no previous psychiatric or neurological history participated in the experiment. 70% (21/30) of volunteers were students of medicine and biology, followed by 20% (6/30) engineering students and 10% (3/30) other students (pursuing degrees in law, business, etc.). The Edinburgh inventory was applied to identify hand laterality (mean ± SD: 0.77 ± 0.18) [49], and the Screening questionnaire for transcranial magnetic stimulation (TMS) was employed to verify if subjects had contraindications to receivingmized sham-contr repetitive TMS (rTMS) [50]. All participants were naive to stimulation. One subject dropped out of the experiment due to pain symptoms during the TMS. Before each experiment, participants were instructed on the design of the experiment.

### 2.2. Experimental Design

**Figure 1:**
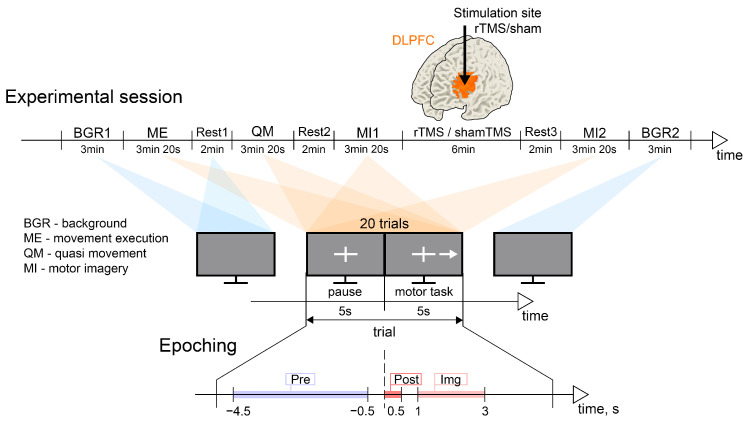
Experimental design. (**Upper panel**) Timeline of the experimental session. (**Middle panel**) The illustration of the typical sequence of the visual cues and the structure of one trial. (**Lower panel**) The illustration of the time intervals of interest within a trial: “Pre” is the baseline pre-que interval [−4.5, −0.5] s; “Post” is the post-cue interval [0, 0.5] s; “Img” is the interval [1, 3] s of motor imagery execution; here, t=0 corresponds to the moment of the appearance of the visual cue to start the movement.

This was a randomized, sham-controlled study. Participants were randomly assigned to receive sham (15 subjects) or real rTMS (15 subjects). These groups were not statistically different with respect to the age and gender of the volunteers: the real rTMS group included 11 females/4 males, age: 22.4 ± 1.3 years; the sham rTMS group included 10 females/5 males, age: 19.9 ± 2.5 years. Throughout the experiment, participants were comfortably seated in a reclining chair with their arms resting on armrests. The experimental scenarios were presented on a 27-inch LCD screen positioned at a distance of 2 m. Participants were instructed to remain relaxed with their eyes open throughout the entire experiment, except when performing specific experimental tasks. The experimental design is illustrated in Figure 1. Prior to and following the experiment, the background EEG activity was recorded for a duration of 3 min. The experiment comprised four distinct tasks: motor execution (ME), quasi-movement (muscle tension can be detected by EMG but is not observed visually, QM), and two instances of motor imagery (MI1 and MI2) involving the dominant (right) hand. The sequence of these tasks was organized to facilitate the accurate execution of kinesthetic motor imagery by the participants. Each experimental task lasted for 3 min and 2 s. A 2 min resting period was provided between tasks. Real or sham rTMS was administered during the interval between the two motor imagery tasks. Following the stimulation, participants were allowed a 2 min rest period before performing the second motor imagery task.

Specific visual cues corresponding to each experimental task were presented on the monitor. Each task consisted of 20 trials, with each trial lasting 10 s (see Figure 1). A trial commenced with the appearance of a white cross on a gray background, serving as a fixation point for 5 s. Subsequently, a right-oriented arrow alongside the cross appeared on the monitor for 5 s, indicating the subject to execute the motor task. Participants were instructed to clench their right hand into a fist during this period, whether in actual execution or in imagination. It was emphasized that participants should refrain from generating EMG activity during MI. No specific instructions were provided regarding the number of overt or imagined movements to be performed. On average, participants managed to execute 2–3 movements during the 5-second interval. A trial concluded with a pause, during which the arrow disappeared from the monitor and the cross reappeared for 5 s. Participants utilized this interval to rest and concentrate on their breathing.

The experimental session lasted for approximately 1.5 to 2 h. Throughout the session, the experimenter continuously monitored muscle activation during quasi-movement and motor imagery tasks using real-time EMG signal monitoring. EEG data were recorded for the entire duration of the experimental session. The participants’ heads remained still during the EEG recording and TMS session. The subjects sat in a special chair with a neck support pillow. This allowed them to relax their neck muscles and perform tasks without moving. However, there were “Rest” sessions that allowed participants to move their heads as well as their bodies.

### 2.3. Experimental Equipment

#### 2.3.1. Electroencephalography Recording

To acquire the data, we employed a 48-channel NVX-52 amplifier (MKS, Zelenograd, Russia). EEG signals were captured using 32 standard Ag/AgCl electrodes positioned in accordance with the international “10-10” system. The earlobe electrodes served as references, while the grounding electrode was positioned on the forehead. Electrode impedance was maintained below 15 kΩ. The EEG signals were digitized at a sampling rate of 1 kHz.

#### 2.3.2. Repetitive Transcranial Magnetic Stimulation

The target region for transcranial magnetic stimulation (TMS) was the left dorsolateral prefrontal cortex (DLPFC) (Figure 1). The specific coordinates of the target site within the DLPFC were set to [79, 46, 51] mm in the CTF coordinate system, referencing the “Colin27” brain MRI averaged template [51]. These coordinates were determined by averaging the reported left DLPFC rTMS seed locations from existing literature [52,53,54]. At the onset of the experimental session, the location of the DLPFC for stimulation was automatically identified on the subject’s scalp using the Localite TMS Navigator system (Localite, Bonn, Germany). To align an individual’s head with the averaged stereotaxic MRI brain atlas, three landmarks (left and right lateral canthi, and nasion) and approximately 20 lines drawn on the surface scalp with a Localite pointer fitted with optical markers were utilized.

The TMS coil was equipped with optical markers, and additional markers were attached to a headband worn on the subject’s forehead to maintain a fixed coil location relative to the head. The position of the coil during stimulation was continuously monitored and recorded.

To induce facilitation of excitability in the DLPFC, we utilized high-frequency rTMS. The stimulation was administered using a focal figure-of-8 coil with an outer diameter of 7 cm for each wing, connected to a standard Neuro MS/D magnetic stimulator (Neurosoft, Ivanovo, Russia). Following the established procedure [50,55,56], the individual resting motor threshold (RMT) was determined as the minimum stimulator intensity required to elicit a peak-to-peak response of at least 50 μV in the flexor digitorum superficialis muscle in a minimum of 5 out of 10 repeated trials. The rTMS parameters were as follows: a duration of 6 min, a frequency of 5 Hz, 1800 pulses, and an intensity set at 90% of the individual RMT. These parameters align with previous experimental studies demonstrating that 5 Hz rTMS over the premotor cortex induces short-lasting (up to 1 h) facilitation in the excitability of the hand area in the primary motor cortex [57,58,59]. Moreover, evidence suggests that high-frequency rTMS at 5 Hz leads to a transient increase in corticospinal excitability [60], with comprehensive reviews available on this topic [61]. The coil was positioned tangentially over the head, with the handle oriented posterolaterally at a 45${}^{\circ }$ angle relative to the sagittal plane. The stimulation protocol adhered to the safety recommendations published in the literature [62,63,64]. Sham stimulation proceeded exactly as described for rTMS, with the coil tilted 90 degrees so that its edge rested on the head. Nowadays, it is a popular method for sham TMS [65,66]. The sham procedure provoked a clicking sound comparable to the real stimulation.

#### 2.3.3. Electromyography Recordings

EMG signals were captured using a pair of COVIDIEN (USA) Ag/AgCl hat-shaped electrodes positioned on the Musculus flexor digitorum superficialis of the right hand. To provide a stable reference, a ground electrode was placed on the left forearm, which exhibited no muscle motion [67]. Electrode impedance was maintained below 15 kΩ. The EMG signal was digitized at a sampling rate of 1 kHz using the NVX-52 amplifier and filtered with a 50 Hz notch filter.

### 2.4. Data Analysis

The data analysis pipeline included the following steps: (1) EEG data preprocessing and epoching (see Section 2.4.1 and Section 2.4.2); (2) sensor-level analysis, including wavelet analysis (Section 2.4.4); (3) estimation of the motor imagery brain response time (Section 2.4.5); (4) source-level analysis (Section 2.4.6); (5) connectivity analysis (Section 2.4.8). EEG data preprocessing, epoching, source-level analysis of data, and the related processing, including source-level statistics, as well as connectivity analysis, were performed in MATLAB 2018b (Mathworks) using FieldTrip Toolbox [68]. Sensor-level analysis and the procedure for estimating the MI brain response time were implemented with the Python MNE package. All other statistical tests and regression analyses were performed using the Python SciPy and NumPy packages. All results were visualized using Matlab or FieldTrip plotting functions and Python packages such as MNE and Matplotlib.

#### 2.4.1. EEG Preprocessing

Power line interference at 50 Hz and its harmonics were removed from the data with a band-stop ([49.5, 50.5] Hz) Butterworth filter. Additionally, a band-pass ([1, 70] Hz) Butterworth filter was applied to reduce the influence of various noise components and physiological artifacts. In the present EEG dataset, low-frequency interference consisted of stray effects and breathing artifacts, while high-frequency interference was associated with muscle artifacts. Since some artifacts, like eye movement and cardiac activity, interfere with the effective frequency range of EEG (1–30 Hz), we used the standard procedure based on an independent component analysis (ICA) to remove these artifacts [69].

#### 2.4.2. EEG Data Epoching

We epoched EEG data relative to the moment of the appearance of the visual cue to start the movement (t=0) and selected the following time intervals of interest (TOIs) within a trial: “Pre”—baseline pre-cue interval [−4.5, −0.5] s corresponding to the pause between two consecutive motor tasks; “Post”—the post-cue interval [0, 0.5] s; “Img”—the interval [1, 3] s of motor imagery performance. We have divided the interval of a motor task performance into two parts, as it is known that the dynamics of brain rhythms fundamentally differ [70]. In the beginning (“Pre” interval), there is a sensorimotor integration process as a reaction to the stimulus, manifested in an increase (synchronization) of the theta rhythm. Then (“Img” interval), there is a decrease (desynchronization) of mu and beta rhythms in the motor and frontal cortex, which accompanies the process of movement execution. Note that “Img” corresponds to the middle part of the post-cue interval, where the motor-related processes should be most pronounced. Also, we considered the intervals Rest2 and Rest3 as the resting-state conditions.

#### 2.4.3. Experimental Conditions

**Figure 2:**
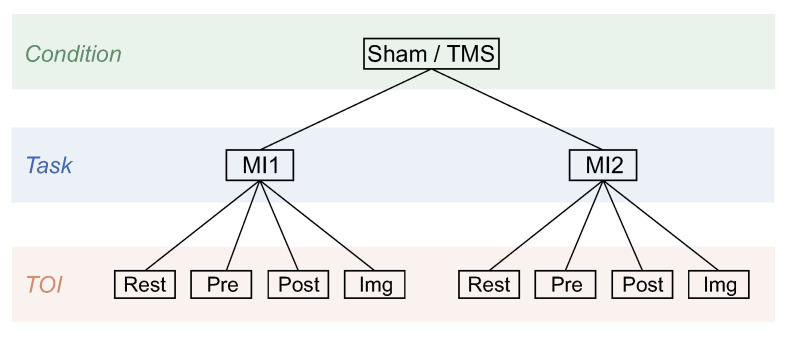
Analyzed in the study conditions, tasks, and TOIs presented in the form of the tree scheme.

In the developed experimental design, there are two experimental conditions (Sham or TMS) and four tasks (ME, QM, MI1, and MI2) with Pre, Post, Img, and resting-state intervals in each in accordance with the recommended terminology [71]. Following the goal, further analysis will focus primarily on analyzing the differences in motor imagery between the TMS and Sham conditions, with the effect of real/sham TMS being evaluated based on comparisons of conditions before (MI1) and after (MI2) real/sham TMS. All conditions, tasks, and TOIs analyzed further in the study are presented in Figure 2 in the form of a tree scheme. Further, we will denote a particular block of data in the following form: $Task_{TOI}^{Condition}$ (e.g., $MI1_{Pre}^{Sham}$).

#### 2.4.4. Sensor-Level Analysis

As a preparation step for the MI brain response time estimation procedure (see Section 2.4.5), we calculated the spectral power distribution over channels in the alpha/mu (10–14 Hz) frequency band in the time interval after the cue ([0, 5] s) using time-frequency analysis implemented in MNE. Particularly, the time-frequency representation of each EEG epoch was obtained via Morlet complex-valued wavelet in the range 10–14 Hz and normalized with baseline (Pre) time interval using “percent” mode, i.e., subtracting the mean of baseline values followed by dividing by the mean of baseline values. The number of cycles in the wavelet transform, *n*, depended on the signal frequency, *f*, as n=f.

#### 2.4.5. Estimation of the MI Brain Response Time

As an objective measure for estimating the rate of MI execution, we used the trials-averaged time of the sensorimotor rhythm desynchronization, which was defined for each subject as the mean moment when the first local minimum of sensorimotor rhythm energy was reached after the cue [72]. We proposed an original algorithm for estimating this characteristic based on alpha-band event-related spectral power analysis. For each subject, we applied the one-sample t-test in a within-trial design with a cluster-based permutation correction [73]. The unit of observation was a time series [0, 4] s of the alpha-band event-related spectral power in the MI task for the given subject. We selected only 13 channels over the motor cortex since we considered the sensorimotor rhythm. The cluster statistic was defined as the sum of all t-values in the cluster. The alpha-threshold for the clusters to be chosen as significant was set to 0.05. Each cluster is constructed from adjacent points with t-values lower than the current threshold. The t-threshold itself varies from the values corresponding to 5%, 2.5%, 1.25%, and 0.15625% alpha levels. For each subject, the test begins with the t-value corresponding to a 5% alpha level. If there were no significant clusters, we repeated the test with the next lower t-value. We applied this procedure because each subject’s desynchronization event is unique and has different properties. Testing with the t-value threshold corresponding to the high alpha level will capture more points since the testing is less sensitive. However, due to this sensitivity, the resulting clusters will likely have higher cluster-alpha values derived from the permutation distribution. Hence, lowering the threshold allows us to get more precise results. However, this also affects the size of the cluster, which will result in more random and meaningless clusters. Thus, the procedure described above helps to capture almost every occurrence of the desynchronization event and reduce the number of nonrelevant clusters.

**Figure 3:**
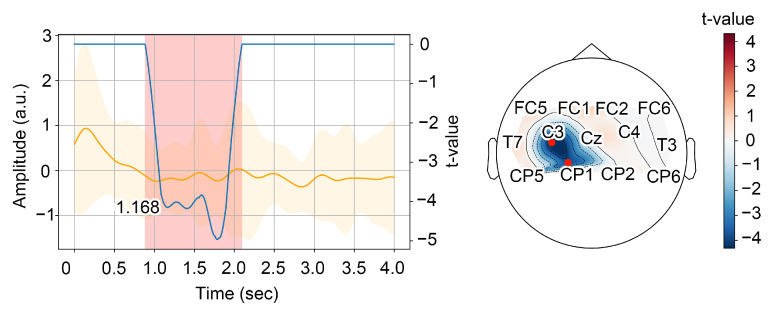
(**Left**) Time series of the normalized energy in the alpha-band averaged over the trials and the significant channels and its dispersion (in orange); time series of the t-value in the first significant cluster averaged over the significant channels (in blue). The red area indicates the time interval corresponding to the first significant cluster; the numerical caption on the graph is the time of the first significant local minimum of the t-values. (**Right**) Topogramm of the t-values averaged over the time interval corresponding to the first significant cluster. The red dots indicate the significant channels.

After obtaining significant clusters, we have chosen the first one as relevant to desynchronization. We considered the first local minimum of the t-value series averaged over significant channels as the mean moment of sensorimotor rhythm desynchronization. To facilitate each event, we plotted every cluster candidate and corresponding topogram and analyzed them manually (see illustration in Figure 3). Based on the analysis of significant desynchronization patterns, we have estimated the trials-averaged time necessary to perform MI.

#### 2.4.6. Source Reconstruction

We analyzed the source power in the predefined time intervals of interest (see Section 2.4.2) in four frequency bands: theta (4–8 Hz), low alpha (8–12 Hz), high alpha (12–14 Hz), and beta (14–30 Hz). These components reflect different aspects of sensorimotor processing [74,75]. We solved the inverse problem and reconstructed source activity from EEG data at each of the predefined points (voxels) in the brain volume, using the exact low-resolution brain electromagnetic tomography (eLORETA) method [76,77,78]. We used the “Colin27” brain MRI averaged template [51] for creating a three-layer (brain, skull, and scalp) head model based on a boundary element method (BEM) [79,80]. The source space consisted of 11,929 voxels inside the brain. The location of the EEG electrodes corresponded to the template head shape.

We re-referenced EEG signals to the common average, subtracted the mean, and filtered with a fourth-order Butterworth $\left[f_{L},f_{H}\right]$-Hz band-pass filter, where $f_{L}$ and $f_{H}$ define the frequency band of interest. Furthermore, we performed time-lock averaging across the epochs of the chosen TOI and computed the covariance matrix. The inverse eLORETA solution yielded estimates of the source power in each voxel, averaged over the selected TOI window for the chosen frequency band.

Finally, we normalized the obtained estimates of the power *P* of each source to the power of the resting-state before the considered task as $nP=\left(P-P_{Rest}\right)/P_{Rest}$ (for Pre, Post, and Img intervals) or to the power of the background EEG activity as $nP=\left(P-P_{BGR1}\right)/P_{BGR1}$ (for Rest1 and Rest2). We used the automated anatomical labeling (AAL) brain atlas [81] to map the location of sources in the anatomical brain regions.

#### 2.4.7. Definition of ROIs

After the group-level statistical evaluation of the source power difference between MI1 and MI2 tasks in different TOIs for the TMS condition, regions of interest (ROIs) were defined based on this effect. It was done to reduce the search space in later analyses (power, correlation, and connectivity analyses). ROIs were defined as dipolar point estimates, separately for each frequency band, that showed the maximal power difference effect (points characterized by the maximum modulo value of the t-statistics).

#### 2.4.8. Connectivity Analysis

Functional connectivity was computed between the previously defined, frequency-specific ROIs for MI1 and MI2 tasks in TMS/Sham conditions, separately for Pre and resting-state intervals. We used the phase-locking value (PLV) [82,83] as a metric of functional connectivity strength defined by the standard formula:

(1) $$PLV_{xy}=\frac{1}{n}\left|\sum_{k=1}^{n}e^{i(\phi_{x,k}-\phi_{y,k})}\right|,$$

where $\phi_{x,k}$ and $\phi_{y,k}$ are the instantaneous phases extracted from the ROI-specific signal estimates x(t) and y(t) via Hilbert transform; *n* is the number of trials. We reconstructed virtual channels in the positions of ROIs to estimate ROI-specific signals. To reconstruct the single-trial virtual channel signals, we multiply the inverse filter for the ROI coordinate that was retrieved from the source reconstruction procedure with the sensor-level EEG signals. Furthermore, we projected the virtual channel time series along the strongest dipole direction. This projection is equivalent to determining the largest (temporal) eigenvector, and we computationally performed it using the singular value decomposition.

### 2.5. Statistical Analysis

The effect of TMS/Sham stimulation on the MI brain response time (MIBRT) and functional connectivity strength between the ROIs was assessed with the Wilcoxon test by comparing $MI1^{TMS/Sham}$ and $MI2^{TMS/Sham}$ conditions with Holmes correction for multiple comparisons.

We compared TOI-averaged source power distributions across conditions in a within-subjects design using a nonparametric permutation test combined with spatial clustering for family-wise error control [73]. Using the Monte-Carlo randomization technique, we performed 16,000 permutations of the condition labels of the individual subjects’ condition-specific source power averages for all dipole locations and used the dependent sample t-statistic. Furthermore, adjacent dipole locations with t-values corresponding to a nominal threshold of $\alpha_{pairwise}$ were grouped into clusters, and their t-values were summed. The null hypothesis was rejected if the maximum cluster statistic in the observed data was in either tail of the permutation distribution (with the critical alpha Bonferroni corrected for multiple comparisons for different frequency bands and TOIs).

Furthermore, for each ROI at a specific frequency, we carried out the Wilcoxon tests in TMS/Sham conditions for MI1 and MI2 tasks because the Shapiro-Wilk test did not confirm the normality of the samples. These tests evaluated if there was a significant difference in the neural activity between MI1 and MI2 in TMS or Sham conditions. The critical alpha level was Holmes-corrected for multiple comparisons. Further, we have also used the Shapiro-Wilk criterion to test the normality of other samples.

The significance of a linear regression between MIBRT and source power change in the ROI between MI2 and MI1 tasks was estimated with the Pearson correlation test implemented in the SciPy package.

## 3. Results

### 3.1. Neural Substrates of MI

**Figure 4:**
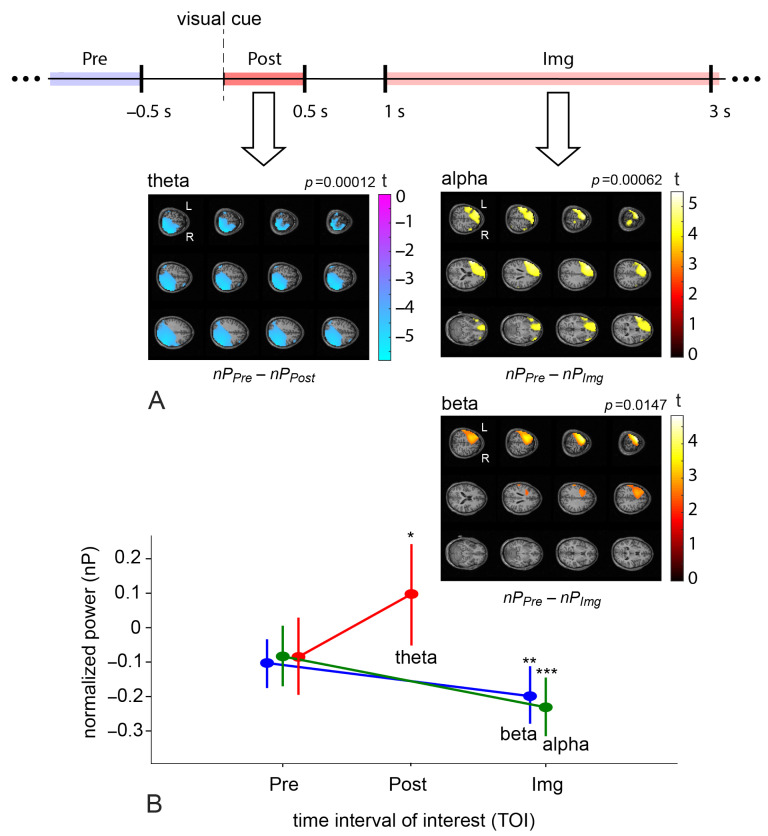
(**A**) The schematic illustration of the time intervals of interest within a trial and normalized source power differences in the first motor imagery task between Pre and Post TOIs ($MI1_{Pre}$ vs. $MI1_{Post}$) for θ-band, Pre and Img TOIs ($MI1_{Pre}$ vs. $MI1_{Img}$) for high α-band and β-band; the data from the TMS and Sham groups were pooled for these statistical tests. Color values indicate t-values at the group level in the revealed negative (for θ-band) and positive (for α-band and β-band) clusters. Values less than 70% of the modulus of the value are masked; *p* is a *p*-value for the cluster corrected for multiple voxel-wise comparisons via the cluster-based permutation test with the Monte-Carlo randomization technique, pairwise α-level equals to 0.02. (**B**) Contrasts of normalized source power (group mean ± standard error (SE)) calculated in the revealed clusters in θ-, α-, and β-bands in the Pre and Post/Img TOIs in MI1 task; ‘*’—p=0.00018, ‘**’—p=0.00075, ‘***’—p=0.00304.

First, we analyzed neural substrates corresponding to MI execution. We contrasted power distributions at the source level between TOIs before and after the visual cue ($MI1_{Pre}$ vs. $MI1_{Post}$) and during MI execution ($MI1_{Pre}$ vs. $MI1_{Img}$) for the MI1 task in the four frequency bands using the nonparametric permutation test. Group statistical evaluation at the cluster level revealed the presence of significant power differences between Pre and Post TOIs in the theta frequency band (p=0.00012) and between Pre and Img TOIs in the high alpha (p=0.00062) and beta bands (p=0.0147). The t-value maps of these contrasts are shown in Figure 4A. The change in cluster-averaged power (see Figure 4B) shows the direction of the effect. The theta power effect is present in the posterior brain area, where power values were higher in the Post TOI compared to the Pre one (see the negative cluster in Figure 4A and the theta-contrast with p=0.00018, t=−4.2 in Figure 4B). This result reflects the typical sensorimotor integration process during a reaction to the cue [70,84,85]. The high alpha and beta power effects exist when comparing Pre and Img TOIs in the left frontal lobe and left motor cortex, including the supplementary motor area; power values were higher in the Pre TOI compared to the Img one (see the positive clusters in Figure 4A and the alpha-contrast with p=0.00075, t=3.7 and the beta-contrast with p=0.00304, t=3.19 in Figure 4B). Such deactivation patterns are due to the process of MI execution and reflect the typical ERD mechanism in the controlateral motor cortex in the mu and beta bands [70,72]. The involvement of the frontal lobe reflects the fact that MI is a complex activity that requires the engagement of high-level cognitive functions, including memory and control [15]. Remarkably, alpha- and beta-clusters include the left DLPFC—the target TMS zone.

### 3.2. Neural Substrates Induced by TMS before MI

**Figure 5:**
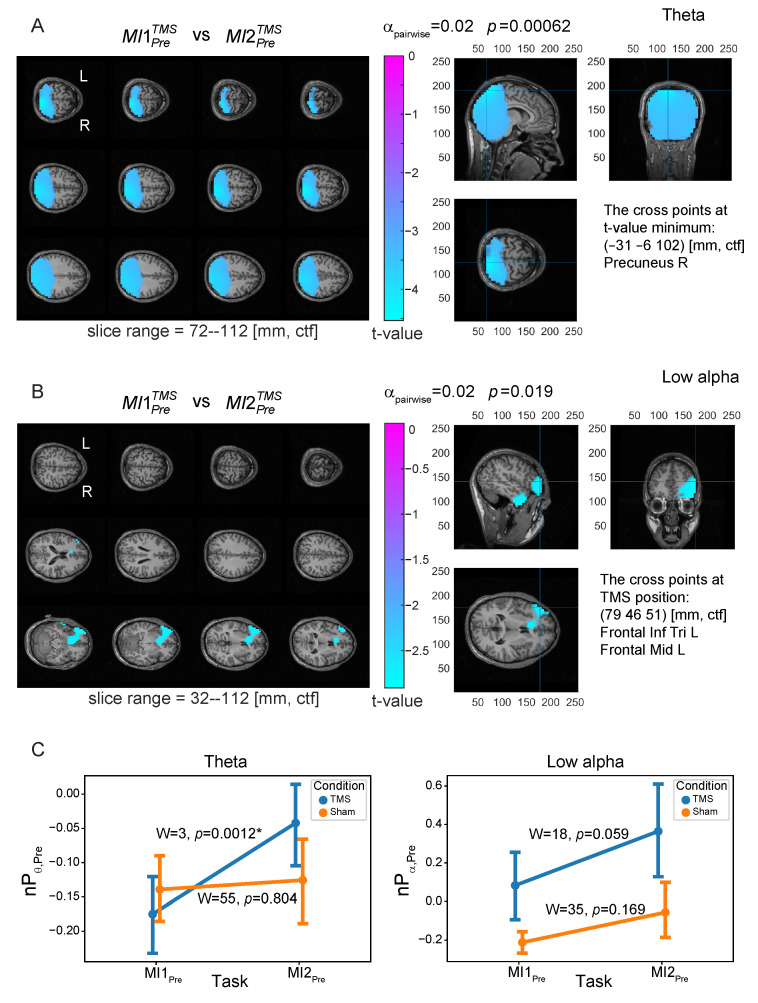
Normalized source power differences in the Pre TOI between the first and the second motor imagery tasks for TMS condition ($MI1_{Pre}^{TMS}$ vs. $MI2_{Pre}^{TMS}$) for (**A**) θ-band and (**B**) low α-band. Color values indicate t-values at the group level in the revealed negative clusters (values greater than 70% of the minimum value are masked); *p* is a *p*-value for the cluster corrected for multiple voxel-wise comparisons via the cluster-based permutation test with the Monte-Carlo randomization technique, pairwise α-level equals to 0.02. The cross at the right panel of (**A**) points at the position of t-value minimum that is located in PrecuneusR brain area; this point was selected as the region of interest for θ-band (ROI1). The cross at the right panel of (**B**) points at the position of TMS target site corresponding to the center of left DLPFC area; this point was selected as the region of interest for low α-band (ROI2). (**C**) Contrasts of normalized source power (group mean ± standard error (SE)) calculated in the selected ROIs in the Pre TOI in MI1 and MI2 tasks for TMS (blue line) and Sham (orange line) conditions; ‘*’ indicates the significant difference at Holmes-corrected significance level.

To reveal neural substrates of TMS influence before and during MI, we contrasted power distributions at the source level between MI tasks before and after TMS ($MI1^{TMS}$ vs. $MI2^{TMS}$) in the four frequency bands for Pre, Post, Img, and Rest TOIs using the nonparametric permutation test (the results are in Table 1). Group statistical evaluation at the cluster level revealed the presence of significant power differences between $MI1_{Pre}^{TMS}$ and $MI2_{Pre}^{TMS}$ tasks for Pre TOI in two frequency bands: theta (p=0.00062, $p_{corr}=0.01$, corrected) and low alpha (p=0.019, $p_{corr}=0.304$, corrected). The t-values maps of these contrasts are shown in Figure 5A,B. The theta power effect is present throughout the bilateral occipital cortex, which extend to the parietal areas with maximal effect in the right precuneus (PrecuneusR) area. In the negative cluster that led to the rejection of the null hypothesis (marked with blue color in Figure 5A), power values were higher in the condition $MI2^{TMS}$ after TMS compared to the condition $MI1^{TMS}$ before TMS. The low alpha power effect is significant only without Bonferroni correction; however, the power difference in the low alpha band demonstrates the importance of discarding it right away. Specifically, the t-values map (Figure 5B) demonstrates the negative cluster in the inferior and middle parts of the left frontal lobe, and the maximal effect was in the area of the TMS target site (left DLPFC). The insignificance (after correction) in the low alpha band is possibly due to the relatively small sample size and large variance in the groups.

Based on these effects, two regions of interest (ROIs) were defined for further analysis (see Table 1): PrecuneusR—in the theta band (ROI1) and left DLPFC—in the low alpha band (ROI2). To control for the revealed power effects induced by TMS, we compared the power in the selected ROIs in the Pre TOI between $MI1_{Pre}$ and $MI2_{Pre}$ for TMS and Sham conditions using the Wilcoxon test (Figure 5C). There was one significant effect in the theta band (ROI1) for the TMS condition (W=3, p=0.0012, corrected), where power was higher after TMS, and no significant effects were observed for the Sham condition. Also, there was a trend toward higher alpha power in ROI2 after TMS. These results prove that the TMS delivered to the left DLPFC results in an increase in theta power in the PrecuneusR area and a tendency to increase alpha power in the DLPFC area. As a consequence, we assume that these ROIs are functionally connected at a resting state. This effect will be considered in detail in Section 3.4.

Note that we analyzed the power ($nP_{\theta ,Pre}$) normalized to the resting-state level, with the normalized theta power being negative in the Pre TOI (see Figure 5C). This means that the theta activity in the Rest TOI is higher than in the Pre TOI. In this way, TMS leads to a decrease in the theta power difference between Rest and Pre TOIs ($nP_{\theta ,Pre}$ approaches 0 from below).

### 3.3. Similarity between Brain Patterns of Sensorimotor Integration and Preactivation with TMS

The TMS-induced pattern discovered in Section 3.2 in theta band with maximal effect in the PrecuneusR area is qualitatively similar to the brain pattern that emerges during the sensorimotor integration process as a result of a reaction to the stimulus. This pattern is shown in Figure 4 (Theta) in the form of the significant negative cluster in the t-values map obtained in the nonparametric permutation test $MI1_{Pre}$ vs. $MI1_{Post}$ in the theta band. This pattern is also characterized by an increase in theta power in a large area of the brain (mostly in the occipital and parietal regions), with a maximum in PrecuneusR zone. Thus, TMS shifts the brain state in the Pre TOI into a state similar to the one that develops as a consequence of reacting to the stimulus before MI execution.

### 3.4. Analysis of Connectivity between ROIs

**Figure 6:**
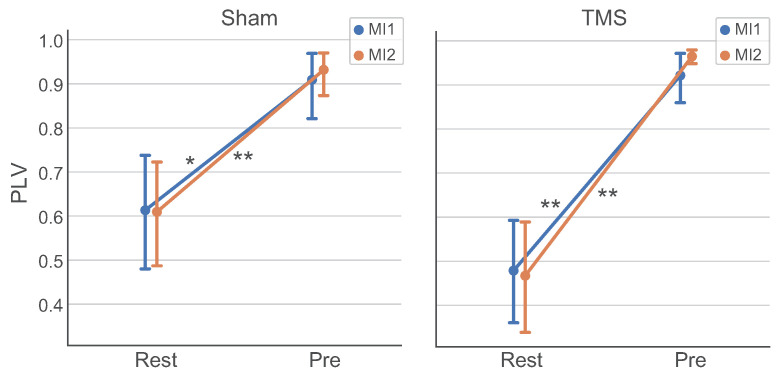
Contrasts of PLVs between PrecuneusR and left DLPFC ROIs (group mean ± SE) in the theta band in the Rest and Pre TOIs for MI1 (blue line) and MI2 (orange line) tasks for Sham and TMS conditions; connections with $p<10^{-3}$ (Holmes-corrected) and $p<10^{-4}$ (Holmes-corrected) are denoted with ‘*’ and ‘**’, respectively.

Using the PLV measure, we have found functional connectivity between the selected ROIs (PrecuneusR and left DLPFC) in the theta band that is significantly stronger in the Pre TOI (PLV>0.9) compared to the Rest TOI (PLV 0.5–0.6). We considered the theta band since this rhythm is usually associated with integration processes in the brain [86,87]. Figure 6 demonstrate the estimated PLVs for the groups in the Rest and Pre TOIs in MI1 and MI2 tasks for TMS and Sham conditions. The carried out statistical comparisons with the Wilcoxon test with Holmes correction have shown the significance of the “TOI” factor ($PLV_{Pre}>PLV_{Rest}$, $p<10^{-4}$) and the absence of the significance of the factors “Task” (p>0.77) and “Condition” (p>0.27). The results indicate that the dynamics of the left DLPFC and PrecuneusR zones show strong phase theta-synchronization in the Pre TOI and weaker synchronization in the Rest TOI, with the level of synchronization independent of the TMS effect.

### 3.5. Effect of TMS on MI Brain Response Time

To analyze TMS influence on the rate of MI execution, we compared the MIBRT between the first and second MI tasks for TMS and Sham conditions using the Wilcoxon test (see Table 2). There was no significant effect on either of the conditions. However, there was a trend towards decreasing MIBRT and its variance after TMS. At this point, we have formulated the suggestion that not all the subjects received sufficient exposure to TMS to achieve a significant change in MIBRT at the group level. We tested this suggestion by conducting a correlation analysis, the results of which are presented in the next section.

Note that it was not possible to estimate MIBRT for all subjects using the algorithm described in Section 2.4.5. We assume that these subjects were unable to successfully perform MI and form a related pattern of brain activity during the entire experiment, so they were excluded from further analysis with MIBRT. This leaves 12 subjects in the TMS group and 11—in the Sham group.

### 3.6. Correlation between MIBRT and Level of Brain Preactivation with TMS

**Figure 7:**
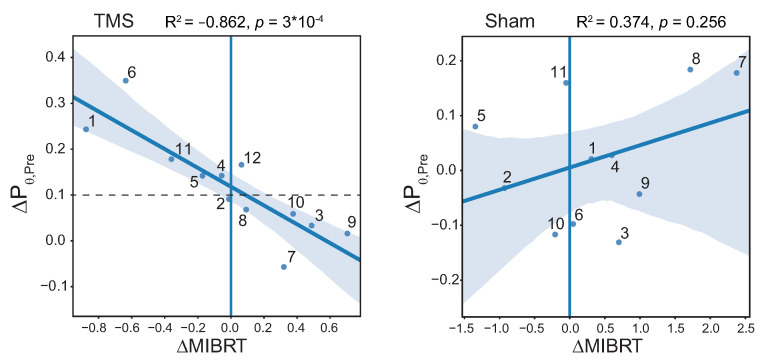
Scatter plots showing the relationships between the change in MIBRT and normalized θ-power change $\Delta P_{\theta ,Pre}$ in the Pre TOI between MI2 and MI1 tasks in the ROI1 brain area for TMS condition (left panel) and Sham condition (right panel); $\Delta P_{\theta ,Pre}=nP_{\theta ,Pre}^{MI2}-nP_{\theta ,Pre}^{MI1}$; $\Delta MIBRT=MIBRT_{MI2}-MIBRT_{MI1}$. The dots denote the individual subjects.

Since we found a strictly significant effect of TMS in the theta band in the PrecuneusR area, we analyzed the correlation between the change in MIBRT and the change in theta power in this ROI between MI2 and MI1 tasks (see Figure 7); sham stimulation was considered as a control as well. As a result, we found a significant negative correlation for TMS condition ($R^{2}=-0.862$, $p=3\times 10^{-4}$ ) and non-significant—for Sham ($R^{2}=0.374$, p=0.256).

For the significant correlation, if the level of brain preactivation with TMS, estimated by the $\Delta P_{\theta ,Pre}$ value, is greater than a certain threshold (∼0.1), then the MIBRT decreases after the TMS (ΔMIBRT<0) session; otherwise, there is either an increase in the ΔMIBRT, or no change. Thus, half of the TMS group (“successful” subgroup) shows a decrease in MI execution time, while the remaining half (“unsuccessful” subgroup) shows an increase.

## 4. Discussion

In this study, we examined the functional contribution of the left dorsolateral prefrontal cortex (DLPFC) to the motor imagery (MI) using excitatory repeated transcranial magnetic stimulation (rTMS).

Stimulation of the left DLPFC influences neural activity in the PrecuneusR leading to s statistically significant increase in the theta-band power on the intervals preceding the MI performing. Based on the connectivity analysis, we highlight the vital role of the functional interaction between these areas. The Phase Locking Value (PLV) shows that functional connectivity between the left DLPFC and the PrecuneusR exists in the resting state and grows on the interval preceding MI (PLV >0.95). During these intervals, subjects focused their attention on the visual cues and prepared for MI execution. Our findings support the recent study that reported a positive causal influence of the left DLPFC on the bilateral precuneus in the resting state [88].

We found a negative correlation between the MI response latency and the precuneus theta-band power. High theta-band power corresponded to the short time between the cue and the formation of the event-related desynchronization. Despite the strong negative correlation between the theta-band power and latency, we did not report a decreased latency in the rTMS group. The mean response latency and its variance declined, but their change was statistically insignificant (Table 2). Detailed analysis indicated that the latency decreased if the theta-band power rose by more than 10% and increased otherwise (Figure 7). Thus, only 50% of participants showed a reduction in MI response latency. One possible explanation is that subjects did not receive adequate exposure to the stimulation of the target brain structure. First, we positioned the TMS coil using an average head model rather than the individual subject’s MRI image. Therefore, some participants were likely to receive activation below the necessary threshold or no activation at all. Second, rTMS susceptibility might vary across subjects, so the duration of stimulation could be insufficient for some of them.

The correlation between MI response latency and theta-band power in PrecuneusR may indicate that preactivation of Precuneus facilitates processing of the visual cue. This hypothesis is supported by the high posterior theta-band power on the post-cue interval, which probably reflects cue processing (Figure 4). Without rTMS, the posterior theta-band power drops on the interval preceding the visual cue when the subjects rest from the previous MI execution and prepare for the next one. This effect may share the mechanisms of the post-movement mu/beta rebound, reflecting an active “clearing-out” of the motor plan and its feedback-based online control [89,90]. The rTMS reduces this drop by providing high theta-band power on the pre-cue interval. We suppose that high pre-cue theta-band power reduces the demands of its activation on the post-cue segment, hence, lowering the latency of MI response.

The obtained results may shed light on the neural interactions behind MI. The literature reports that the left DLPFC is a hub of the fronto-parietal central executive network (CEN) involved in executive functions [91]. Stimulation over the DLPFC is an efficient modulator of both neural activity and general cognitive performance [92,93]. Precuneus belongs to the default mode network (DMN), a task-negative network that subserves introspection and social cognition [94,95,96,97]. Usually, task performance requires the deactivation of DMN and activation of CEN, which shift attention from internal thoughts and feelings to the external world and stimuli [88,98,99]. In line with our results, a recent meta-analysis by Hardwick et al. revealed consistent recruitment of the left DLPFC in the MI task [15]. Glover et al. discussed the contribution of DLPFC to the frontal-executive functions in MI [100]. Participating in the different functional networks, left DLPFC and PrecuneusR interact underlying many cognitive functions [88]. For example, cognitive task switching requires strengthening positive functional coupling between the left DLPFC and the bilateral Precuneus [91]. Thus, we suppose that the coupling between the left DLPFC and PrecuneusR subserves the dynamic interaction between CEN and DMN in the MI task.

These networks may interact as follows. The rTMS targets the left DLPFC and activates task-positive CEN. Strengthening connectivity between the DLPFC and PrecuneusR during the pre-cue interval deactivates task-negative DMN. Other studies support this hypothesis by reporting a negative correlation of the theta-band power with the DMN activation [101,102]. In our study, increased theta-band power in PrecuneusR may also indicate DMN deactivation. Sauseng and colleagues related high theta-band frontoparietal coherence with the increased demands on central executive functions in working memory [103]. MI also involves working memory functions; therefore, it activates CEN and induces theta-band power. We suggest that modulating DLPFC TMS may turn off the DMN, providing a transition from an internally focused mental state to a mental state focused on an external task. This latter speeds up cue processing and reduces the overall response latency.

Recently, researchers reported the ability of rTMS to modulate functional connectivity [92,104,105]. The high-frequency rTMS over the right DLPFC provoked an alteration of the prefrontal-hippocampal interaction during the working memory task [106]. The inhibitory rTMS over the left DLPFC reduced its connectivity with the brain regions across the DMN [65,107]. Some studies also confirmed alternating resting-state DMN activity after the inhibitory rTMS of the DLPFC [92,108].

Here, we applied excitatory rTMS and expect to facilitate functional connectivity. The rTMS may affect brain activity through different mechanisms. First, rTMS at frequencies of 5 Hz or higher enhances cortical excitability [109,110]. Second, 5 Hz rTMS may modulate the natural brain rhythm, defining a resonance with theta activity [41,111]. Both mechanisms may cause the growth of the theta (4–7 Hz)-band power in our study.

It is widely accepted that training in MI-based brain-computer interface (BCI) is an effective approach in neurorehabilitation therapy for people with impaired motor functions, such as patients with tetraplegia, spinal cord injury, and brain injuries like stroke or amyotrophic lateral sclerosis [112,113,114]. Many studies have shown that integrating BCI control into exoskeleton-assisted physical therapy can improve the post-stroke rehabilitation process [115,116]. Considerable efforts have been made to include feedback on different modalities in MI-based BCI systems to improve performance and promote subject motivation and engagement in training, which can increase the effectiveness of rehabilitation procedures [7,117,118]. In this perspective, we see two main directions of application of the revealed TMS-induced effects in modern neurorehabilitation protocols: (1) accelerating motor imagery, learning and improving its quality by preliminary stimulation of the DLPFC; (2) using it to implement feedback.

Several limitations exist in this study. One was the lack of behavioral measures, making it impossible to relate the imaging findings to behavioral consequences. We overcome this limitation to some extent by developing an algorithm for estimating motor imagery brain response time by ERD formation latency. Another limitation is the absence of individual MRIs of the subjects. We positioned the TMS coil over the left DLPFC using a neuro-navigation system based on the MRI average head model brain. So, we cannot ascertain that we stimulated the exact location of the left DLPFC for all subjects. Other limitations include the small sample size and the across-subject design, which may contribute to large inter-subject variations.

## 5. Conclusions

The motor imagery (MI) tasks incorporate processing the visual cue and performing motor imagery recruiting different brain networks. Previous studies reported activation of the central executive network (CEN) to utilize working memory and deactivation of the default mode network (DMN) to focus attention on the visual cue. Using repetitive transcranial magnetic stimulation (rTMS), we excited the left dorsolateral prefrontal cortex (DLPFC), the essential node of CEN, and observed high theta-band power in the PrecuneusR, a part of DMN. Connectivity analysis revealed strong synchronization of the neural activity in DLPFC and PrecuneusR during the MI task compared to the rest-state. The PrecuneusR theta-band power was negatively correlated with the time spent between the visual cue onset and mu-band desynchronization (ERD), a biomarker of MI. We suppose that DLPFC coordinates the interaction between CEN and DMN. It may turn off DMN to facilitate focusing attention on the visual cue, speeding up its processing and lowering the latency of ERD formation.

## Figures and Tables

**Table 1:** Results of the group-level statistical comparisons of source power distributions between MI tasks before and after TMS ($MI1^{TMS}$ vs. $MI2^{TMS}$) in the four frequency bands for Pre, Post, Img, and Rest TOIs. Here, *p* denotes the *p*-value estimated in the nonparametric permutation test; $p_{corr}$ denotes the *p*-value Bonferroni-corrected for multiple comparisons; “n.s.” is non-significant. Column ROI includes locations of regions of interest and their names per frequency and TOI defined in the statistical testing. For compactness, we omit the “TMS” designation in the records of all compared conditions.

Frequency Band	Condition 1	Condition 2	Significance	ROI,
				CTF Coordinates (mm)
theta	Rest2	Rest3	n.s.	–
	$MI1_{Pre}$	$MI2_{Pre}$	p=0.00062,	PrecuneusR,
			$p_{corr}=0.01$	(−31, −6, 102)
	$MI1_{Post}$	$MI2_{Post}$	n.s.	–
	$MI1_{Img}$	$MI2_{Img}$	n.s.	–
low alpha	Rest2	Rest3	n.s.	–
	$MI1_{Pre}$	$MI2_{Pre}$	p=0.019,	left DLPFC,
			$p_{corr}=0.304$	(79, 46, 51)
	$MI1_{Post}$	$MI2_{Post}$	n.s.	–
	$MI1_{Img}$	$MI2_{Img}$	n.s.	–
high alpha	Rest2	Rest3	n.s.	–
	$MI1_{Pre}$	$MI2_{Pre}$	n.s.	–
	$MI1_{Post}$	$MI2_{Post}$	n.s.	–
	$MI1_{Img}$	$MI2_{Img}$	n.s.	–
beta	Rest2	Rest3	n.s.	–
	$MI1_{Pre}$	$MI2_{Pre}$	n.s.	–
	$MI1_{Post}$	$MI2_{Post}$	n.s.	–
	$MI1_{Img}$	$MI2_{Img}$	n.s.	–

**Table 2:** The results of the group-level statistical comparisons with the Wilcoxon test of MIBRT between MI tasks before and after sham or real TMS ($MI1^{Sham/TMS}$ vs. $MI2^{Sham/TMS}$) and the values of group mean MIBRT ± SE for different conditions. Here, $p_{corr}$ denotes the *p*-value Holmes-corrected for multiple comparisons.

Condition	Task 1/Group Mean	Task 2/Group Mean	W-Value	$p_{\mathrm{corr}}$
	MIBRT ± SE, s	MIBRT ± SE, s		
Sham	MI1/1.56 ± 0.28	MI2/1.69 ± 0.26	21	0.64
TMS	MI1/1.36 ± 0.18	MI2/1.18 ± 0.14	37	0.91

## Data Availability

The data presented in this study are available on request from the corresponding author.

## Abbreviations

The following abbreviations are used in this manuscript:

EEG	Electroencephalogram
DLPFC	Dorsolateral prefrontal cortex
MI	Motor imagery
rTMS	repetitive Transcranial magnetic stimulation
M1	Primary motor cortex
SMA	Supplementary motor area
PMd	Dorsal premotor cortex
CEN	Central executive network
DMN	Default mode network
ERD	Event-related desynchronization
SD	Standard deviation
ME	Motor execution
QM	Quasi-movement
EMG	Electromyography
TOI	Time interval of interest
ROI	Region of interest
RMT	Resting motor threshold
MIBRT	MI brain response time
ICA	Independent component analysis
PLV	Phase-locking value
BCI	Brain-computer interface
